# A Review of Organophosphate Esters with Different Functional Groups: Focusing on Microbial Degradation

**DOI:** 10.3390/toxics14070618

**Published:** 2026-07-15

**Authors:** Jiani Zhang, Yangmei Fei, Shu Huang, Yang Li, Yujiao Cui, Jiahong Li, Shuo Li, Xiaotong Wang, Jiaxin Shi, Fanlong Kong

**Affiliations:** 1 College of Earth Science and Engineering, Shandong University of Science and Technology, Qingdao 266590, China; 2 School of Environment and Geography, Qingdao University, Qingdao 266071, China; 3 Carbon Neutrality and Eco-Environmental Technology Innovation Center of Qingdao, Qingdao 266071, China; 4 Shandong Key Laboratory of Synergistic Control of Complex Multi-Media Pollution, Qingdao 266237, China

**Keywords:** organophosphate esters, microbial degradation, environmental fate, biodegradation pathways, ecological risk assessment

## Abstract

Organophosphate esters (OPEs), characterized by diverse chemical substituents, have emerged as widely used flame retardants and plasticizers, replacing polybrominated diphenyl ethers (PBDEs) under global regulatory actions. However, due to their environmental persistence, bioaccumulation potential, and multiple toxic effects, OPEs are now recognized as emerging pollutants and have attracted extensive research attention. This review systematically compares structurally distinct OPEs in terms of their environmental occurrence, physicochemical properties, mobility, and biodegradation fate. We place special emphasis on recent advances in microbial degradation and enzymatic transformation pathways under both aerobic and anaerobic conditions, with a focus on key degrading strains, metabolic intermediates, and underlying mechanisms. Furthermore, factors influencing biodegradation rates (including compound structure, microbial community composition, and environmental variables) are comprehensively examined. By identifying critical research gaps and proposing future directions, this review aims to provide a scientific foundation for sustainable management and effective risk assessment of OPEs in the environment.

## 1. Introduction

Organophosphate esters (OPEs) are synthetic chemicals widely used as primary components in flame retardants and plasticizers [1]. Initially, OPEs were regarded as “environmentally friendly” additives due to their low persistence, minimal environmental transport, and low bioaccumulation potential [1]. Consequently, with the phase-out of polybrominated diphenyl ethers (PBDEs) under the Stockholm Convention, OPEs were adopted as suitable substitutes [2]. Global consumption of OPEs has increased significantly since the 1940s, rising from 620,000 tons per year in 2013 to 2.8 million tons in 2018, with an estimated 3.1 million tons in 2023 [3,4]. This massive consumption encompasses a variety of high-production-volume compounds. Representative examples include tris(2-butoxyethyl) phosphate (TBOEP), tris(n-butyl) phosphate (TNBP), and triphenyl phosphate (TPHP), which have been historically predominant in European and North American markets, as well as chlorinated OPEs such as tris(2-chloroethyl) phosphate (TCEP) and tris(1,3-dichloro-2-propyl) phosphate (TDCIPP), which have seen extensive use in Asia, particularly in China [5,6].

As a result, OPEs are physically incorporated into materials during production and application, rendering them vulnerable to environmental release through abrasion, volatilization, and leaching [7]. Consequently, OPEs have been ubiquitously detected across diverse environmental media, including natural freshwater, seawater, indoor and outdoor air, sediments, and soil, as well as in biological samples such as aquatic organisms, human blood, and human placenta [8,9]. Critically, OPEs exhibit intrinsic properties typical of persistent organic pollutants: they are resistant to degradation, capable of long-range environmental transport, and possess bioaccumulative potential [10,11]. These properties underpin their widespread distribution and lead to sustained exposure. Such exposure is linked to adverse health effects, including neurotoxicity, reproductive toxicity, and endocrine disruption [12]. Therefore, given their pervasive environmental occurrence and documented toxicity, there is an urgent and growing need to develop effective strategies to mitigate the potential risks OPEs pose to ecosystem integrity and public health.

Microbial degradation is considered an economical and environmentally friendly method for mitigating OPEs pollution [13]. Laboratory studies have demonstrated biodegradation pathways for various OPEs, yet a systematic understanding of how these pathways differ across OPEs categories remains lacking. Based on the substituent structure of ester bonds, OPEs can be categorized into chlorinated (Cl), alkyl, and aryl types [14]. *Burkholderiales* and *Rhizobiales* represent dominant bacteria that were enriched in TCEP microcosms and were capable of TCEP degradation [15]. *Sphingomonans*, *Nocardioides*, and *Streptomyces* were the crucial contributors to the degradation of TNBP in anoxic soils [16]. Different from aerobic degradation with P-O bond cleavage, C-O bond cleavage was certified as the mode of removal a butyl side chain for TNBP to produce dibutyl phosphate (DNBP) during the anaerobic microbial degradation [16]. Recently, *Klebsiella* was identified to degrade nonhalogenated OPEs, including TBOEP, TNBP, and TPHP, under aerobic conditions by sludge cultures from a conventional sewage plant [17]. Despite these isolated findings, comprehensive analyses of biodegradation pathways and intermediates for different OPE categories remain very limited.

This review aims to systematically elucidate the environmental behavior and microbial degradation mechanisms of Cl-, alkyl-, and aryl-OPEs, with an emphasis on key factors governing the degradation process. Given the structural similarities within each OPE class, their microbial degradation pathways and associated enzyme systems exhibit class-specific patterns. Understanding these structure–enzyme relationships provides a basis for predicting the degradation potential of structurally analogous novel OPEs (NOPEs).

## 2. Classification and Properties of OPEs

**Figure 1:**
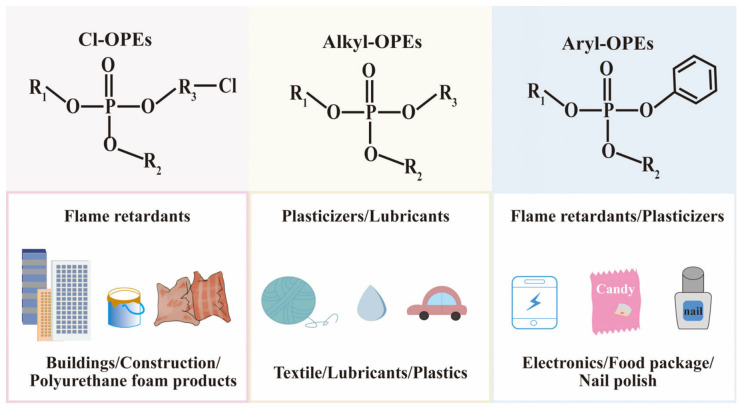
Representative chemical structures and major applications of Cl-, alkyl-, and aryl-OPEs.

With the acceleration of industrialization and the growing demand for flame retardants, the production and consumption of OPEs have gradually increased worldwide (Figure 1) [17]. The fundamental molecular structure of OPEs is represented as O=P(OR)_3_ or S=P(OR)_3_, where R denotes various substituent groups attached to the main molecular chain [18]. Based on the substituent structure of ester bonds, OPEs can be categorized into Cl-, alkyl-, and aryl-OPEs [14]. Despite sharing a common phosphate backbone, these three classes exhibit distinct physicochemical properties due to their specific substituents (Table S1) [14,18].

The characteristic molecular structure of Cl-OPEs lies in the substitution of the phosphate backbone with chlorine atoms [19]. These chlorine atoms replace hydrogen atoms on alkyl or alkene chains via covalent bonds, forming compounds such as TCEP, tris(1-chloro-2-propyl) phosphate (TCIPP), and TDCIPP [20]. Various synthesis approaches for Cl-OPEs in industries have established them as synthetic substances. For instance, TCEP can be synthesized from 2-chloroethanol with phosphorus oxychloride Equation (1), phosphoric acid, ethylene with chlorine Equation (2), and ethylene oxide with phosphorus oxychloride Equation (3).

3ClCH_2_CH_2_OH + POCl_3_ → (ClCH_2_CH_2_O)_3_PO + 3HCl (1)

H_3_PO_4_ + 3C_2_H_4_ + 3Cl_2_ → (ClCH_2_CH2O)_3_PO + 3HCl (2)

3CH_2_CH_2_O + POCl_3_ → (ClCH_2_CH_2_O)_3_PO (3)

The annual production of TCEP, TCIPP, and TDCIPP increased from fewer than 14,000 tons in 1986 to 38,000 tons in 2012 in North America [14]. Additionally, as early as 2006, the annual production of TDCIPP in the United States reached 22,700 tons and has continued to rise [21]. In contrast to alkyl- and aryl-OPEs, Cl-OPEs were primarily developed as alternatives to brominated flame retardants (BFRs) for various domestic and industrial applications [22]. The presence of chlorine atoms enhances their flame-retardant properties, with effectiveness increasing proportionally to halogen content [14,23]. Therefore, Cl-OPEs have become key large-scale flame retardants used in coatings, textiles, floor polishes, latex, and construction materials [8,20,24]. However, the use of TCEP and TDCIPP has been explicitly prohibited in the manufacture of children’s products and residential upholstered furniture in the United States [17]. Tris(2-chloro-1-methylethyl) phosphate (TCPP) serves as a suitable replacement, being cost-effective and readily available, and acts as a major building block and flame retardant additive in synthetic flexible polyurethane (PU) foams (comprising more than 98% TCPP) [25]. Therefore, the development of green and efficient degradation pathways for TCPP has become a critical step in controlling its environmental release and mitigating the substitution risk associated with this emerging contaminant.

Alkyl-OPEs primarily consist of TBOEP, TNBP, tri(2-ethylhexyl) phosphate (TEHP), and triethyl phosphate (TEP) [26]. The industrial synthesis of alkyl-OPEs generally involves esterification reactions. For instance, TBOEP is produced by reacting alcohol ether materials with phosphorus oxychloride in the presence of catalysts [27].

H_3_PO_4_ + ROH → H_2_PO_4_OR + H_2_O (4)

H_2_PO_4_OR + ROH → HPO_4_(OR)_2_ + H_2_O (5)

HPO_4_(OR)_2_ + ROH → PO_4_(OR)_3_ + H_2_O (6)

In the United States, the annual production of TBOEP ranged from 450 to 4500 tons in 2012, while global production estimates for TBOEP were approximately 5000–6000 tons [28,29]. In addition, alkyl-OPEs are widely used as plasticizers in synthetic rubber, plastic products, and textiles, where they enhance transparency and UV resistance [9,30,31].

Compared to short-chain alkyl-OPEs, long-chain alkyl-OPEs demonstrate higher molecular mass and hydrophobicity [3]. Consistent with this trend, TNBP and TBOEP exhibit low water solubility (0.28 g/L and 1.2 g/L, respectively) and high octanol–water partition coefficient (log Kow) value (4.0 and 3.75, respectively) [32,33,34]. Therefore, their environmental persistence, including resistance to conventional removal methods and potential for bioaccumulation, has led to widespread detection in aquatic environments (including surface water, groundwater, and drinking water), raising significant concerns [34].

The distinguishing structural feature of aryl-OPEs is the substitution of aryl rings for alkyl chains on the phosphate ester skeleton [35]. This leads to a structural spectrum ranging from the simple, symmetric TPHP, to the bridged bisphenol A bis (diphenyl phosphate) (BDP), and finally to mixed-substitution analogues like cresyl diphenyl phosphate (CDP) and 2-ethylhexyl diphenyl phosphate (EHDPP) [36]. Aryl-OPEs are typically synthesized through nucleophilic aromatic substitution (SNAr) reactions, where phenolic compounds (PhOH) react with phosphorus oxychloride (POCl_3_) to form O-phosphate ester bonds [37]. For instance, resorcinol diphosphate (RDP) can be preferentially synthesized using resorcinol, phenol, and phosphorus oxychloride as raw materials, with MgCl_2_ serving as a catalyst [37]. Aryl-OPEs have gained prominence as halogen-free flame retardants, replacing halogenated OPEs in numerous consumer products following regulatory restrictions [38]. They are extensively employed as plasticizers, lubricants, and flame retardants across diverse sectors including electronics, pigment dispersions, and food packaging [36,38,39]. A prime example is TPHP, which is commonly incorporated into mobile phone screens, nail polish, cable insulation, and circuit boards [40,41]. Reflecting its widespread use, the annual production of TPHP in the United States ranged from 4500 to 22,700 tons in the late 1990s and has seen a substantial increase since 2005 [42]. Similarly, 2-Ethyl-Hexyldiphenyl Phosphate (EHDPP) is prized for its flexibility, durability, and flame-retardant properties, serving as a key component in fireproof coatings and food packaging [43]. Correspondingly, its production volume in the United States has reached 1–10 million pounds annually since 1986, a trend mirrored in Europe [44]. Beyond these, tricresyl phosphate (TCP) finds major application not as a flame retardant, but primarily as an anti-wear and anti-abrasive additive in industrial fluids such as hydraulic oils, engine lubricants, and greases [45,46]. In contrast to Cl-OPEs, aryl-OPEs, along with alkyl-OPEs, are also extensively employed as plasticizers across various sectors, further highlighting the functional divergence between these chemical classes [47].

The environmental distribution, fate, and toxicity of OPEs were fundamentally governed by their distinct molecular structures and the resulting physicochemical properties, which vary systematically among Cl-, alkyl-, and aryl-OPEs.

Cl-OPEs are characterized by a hydrophilic phosphate group and three hydrophobic haloalkane groups, resulting in predominantly lipophilic behavior [48]. This combination may enable them to persist in the aqueous phase and sediment porewater, leading to prolonged environmental retention and sustained bioavailability to aquatic organisms [18]. In contrast, alkyl-OPEs and aryl-OPEs exhibit greater hydrophobicity, resulting in a stronger partitioning affinity for organic matter in sediments and soils [32]. The hydrophobicity (log Kow) of all OPEs facilitates long-range environmental transport and correlates directly with their bioaccumulation potential [1]. Notably, aryl-OPEs, owing to their elevated log Kow, demonstrate pronounced cellular uptake and consequently higher cytotoxicity [3]. For human exposure routes such as dermal contact, alkyl- and Cl-OPEs show rapid skin absorption and penetration (57.6–127% of the applied dose), a process inversely related to molecular mass [49].

In summary, alkyl-OPEs are highly hydrophobic and soil/sediment-bound, Cl-OPEs are persistent, water-mobile, and bioavailable, while aryl-OPEs are characterized by high hydrophobicity, strong bioaccumulation, and significant cytotoxicity. The representative structures of each OPEs class are illustrated in Figure 1, and a comprehensive summary of their physicochemical properties and environmental occurrence is provided in Table S1. This systematic understanding of their physicochemical drivers is essential for accurate risk assessment and the development of targeted remediation strategies.

## 3. Environmental Occurrence and Toxicity

### 3.1. Major Sources and Emission Pathways

**Figure 2:**
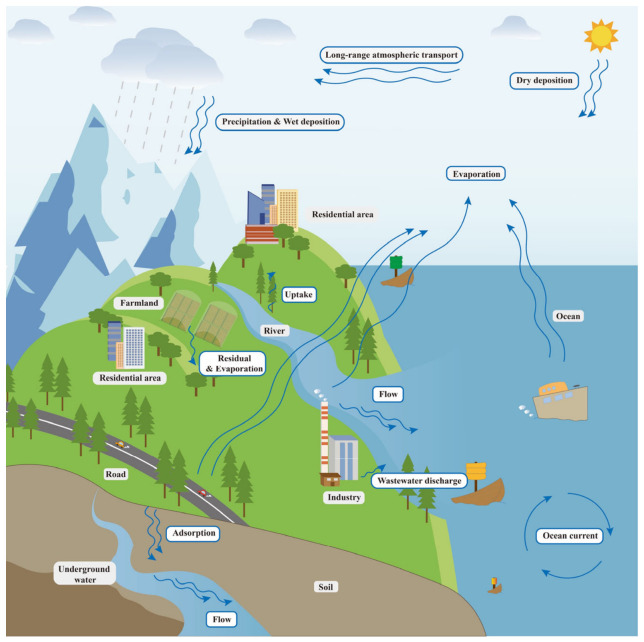
Potential sources and fate of OPEs in the environment. OPEs flow from the primary producer to the commercial user to the consumers to disposal. Atmospheric and aqueous escape releases at each step. Arrows indicate transport pathways (e.g., atmospheric deposition, river flow, wastewater discharge, and ocean currents).

As exclusively anthropogenic synthetic chemicals, OPEs enter the environment solely through human activities related to their production, use, and disposal [50]. These release pathways can be systematically categorized into three major sectors based on emission characteristics and scale: industrial point sources, diffuse agricultural-aquacultural sources, and transportation-mediated releases (Figure 2) [51].

Industrial and municipal point sources represent the dominant concentrated emissions and often form localized pollution hotspots. The primary release pathways stem from the use and end-of-life treatment of OPEs-containing consumer and industrial goods, such as electronics, furniture, construction materials, and vehicles [52,53]. E-waste recycling sites represent critical pollution hotspots, with soil OPEs concentrations reaching as high as 2,120,000 ng/g in Guiyu, China, and dust levels ranging from 34,000 to 270,000 ng/g in Canadian dismantling facilities [54,55]. Airborne exposure at these sites is also significant, with TPHP levels of 850 ng/m^3^ reported in Finnish dismantling facilities [55]. Similarly, end-of-life vehicle (ELV) processing facilities represent significant hotspots, particularly for Cl-OPEs, where sediment concentrations as high as 3.0 × 10^4^ mg/kg have been recorded [56]. In addition, wastewater treatment plants (WWTPs) act as critical continuous emission sources, where incomplete removal (e.g., only 49% degradation reported) results in substantial OPEs discharges into receiving waters [57,58].

In contrast to concentrated point sources, diffuse agricultural sources, particularly plastic film application (2.53 million tons annually worldwide, containing 5.38–115 μg/kg OPEs), contribute to widespread soil contamination with median total OPEs exceeding 228 ng/g [59]. Aquaculture systems are additionally impacted through contaminated equipment and animal feeds, with fish feed OPE levels (46.0 ng/g dw) substantially exceeding those in surrounding sediments [60]. Once introduced via these pathways, OPEs can accumulate in aquatic biota and undergo biomagnification through the food web, thereby initiating a cascade of trophic transfer that represents a latent yet pressing concern for long-term ecosystem integrity and food security [60,61].

Transportation and associated mobilization constitute a third distinct sector, characterized by direct emissions and secondary releases linked to mobility. Vehicular activity is a key contributor, with both exhaust and non-exhaust emissions (e.g., from tire and brake wear) releasing OPEs [10,62]. This link is demonstrated by an exponential increase in OPEs concentrations in road dust with rising traffic density, where specific compounds like TEP and Tri-n-butyl phosphate (TBP) have even been proposed as chemical tracers for gasoline vehicle emissions [10]. Concurrently, marine transportation and port operations have been identified as significant sources in coastal environments. Emissions from fuels, lubricants, potential spills, and routine port activities release OPEs, which have been traced as notable contributors in systems such as the Bohai Sea and the Yangtze River Estuary [63,64].

### 3.2. Environmental Mobility and Distribution of OPEs

The environmental mobility and ultimate fate of OPEs are governed by their interactions across atmospheric, aquatic, and sedimentary compartments, with transport potential varying significantly among different structural classes.

Atmospheric transport facilitates long-range dispersion of OPEs, particularly Cl-OPEs, which exhibit greater long-range atmosphere transport (LRAT) potential than alkyl- and aryl-OPEs due to their volatility and persistence against OH-radical degradation [11,65,66,67]. Atmospheric deposition serves as a critical secondary input, with “mountain cold-trapping” accumulating OPEs in glaciers (Cl-OPEs accounting for 66% of glacial burden) [68,69], and air–sea exchange strongly favoring net deposition into oceans [70]. This highlights that atmospheric transport serves as a crucial and persistent external source of OPEs in the ocean, dominating the input flux of this pollutant to the open ocean.

The aqueous mobility of OPEs, defined as the fraction of a compound that enters the water column and is subsequently transported via advection, without being immobilized through sorption or degraded, governs their long-distance dispersal through rivers and oceans [71]. Notably, approximately 50% of OPEs can survive conventional water treatment processes, and the aqueous half-lives of Cl-OPEs are particularly prolonged, ranging from 121 to 212.5 days, which exceed the persistence criteria established by the Stockholm Convention [58,65]. This inherent resistance to degradation ensures their widespread detection in global aquatic systems. Riverine runoff serves as the primary vector transporting terrestrial OPEs to oceans, with estimates of 16 tons annually entering the Bohai Sea via major rivers [72]. Salinity gradients play a crucial modulating role, particularly enhancing the desorption of Cl-OPEs from sediments and thus promoting their aqueous mobility in estuarine zones [12,73]. Ocean currents further redistribute OPEs, with transport efficiency most significant at intermediate depths (500 m) [74].

Sediments serve as significant sinks and long-term reservoirs for OPEs in the environment [68]. The occurrence and transformation of OPEs in sediments are jointly regulated by their physicochemical properties and sediment characteristics, which can be analyzed from two main aspects: storage stability and partitioning behavior. In terms of storage stability, the persistence of OPEs varies significantly depending on their structural classes. Cl-OPEs exhibit the longest half-lives (654–1621 days), whereas alkyl- and aryl-OPEs generally have much shorter half-lives (78–338 days) [68]. This difference is closely linked to their adsorption–desorption behavior in sediments. The adsorption process of OPEs typically follows three stages: rapid adsorption, slow adsorption, and final equilibrium [75]. Their desorption potential largely depends on their binding states in sediments, with average desorption ratios of loosely bound, stabilized adsorbed, and tightly bound OPEs reaching approximately 86.21%, 73.41%, and 43.01%, respectively [75]. This result suggests that the longer half-lives of Cl-OPEs are likely associated with their tendency to form binding states with lower desorption rates, thereby enhancing their stability in sediments. Sediment properties such as specific surface area and porosity influence the binding mechanisms of OPEs, including electrostatic interactions and covalent bonding [75,76].

Regarding sediment–water partitioning and spatial distribution, the distribution behavior is primarily controlled by organic carbon content (log P-Koc) and log Kow [77]. Compared to alkyl- and Cl-OPEs, aryl-OPEs exhibit the highest hydrophobicity, showing the greatest tendency to establish partitioning equilibrium between water and sediment [77]. Consequently, they are preferentially enriched in sediments, contributing to approximately 47% of the total OPEs burden in marine sedimentary environments [68,78]. The spatial variability of OPEs is further influenced by both their inherent properties (log Kow, solubility, vapor pressure) and sediment characteristics (grain size, nutrient content, dissolved inorganic nitrogen (DIN), total nitrogen (TN)) [12,79]. Due to their generally high log Kow values, many OPEs are lipophilic and have a strong tendency to bind to carbon-rich suspended particulate matter, eventually accumulating in sediments, although some others, typically the chlorinated ones, may exhibit lower log Kow and higher water solubility [80].

In deep marine ecosystems, the long-range transport and ultimate fate of OPEs are driven by complex mechanisms. Their vertical transport primarily follows a “solvent switching” mechanism, whereby OPEs first adsorb onto particulate organic matter (POM) at the ocean surface and then undergo vertical transport via POM settling [74]. During this process, highly hydrophobic and high-molecular-weight compounds gradually partition into deeper water layers and sediments, leading to increasing concentrations with depth-a phenomenon known as “solvent depletion” [79]. This ultimately results in a filtering effect at the sediment–water interface. Notably, horizontal transport also plays a crucial role, as demonstrated by ocean currents contributing over 90% of the total OPEs load found in sediments of the Okinawa Trough [79].

In summary, the environmental distribution of OPEs was a function of their intrinsic physicochemical properties (volatility and hydrophobicity) interacting with compartment-specific processes (LRAT, riverine discharge, sedimentation, biotic uptake). This integrated multi-media transport framework ultimately determines their regional dispersion, long-range delivery to pristine environments, and long-term ecological sequestration.

### 3.3. Potential Toxicity of OPEs

Toxicity studies consistently show that Cl-OPEs and aryl-OPEs exhibit greater potency than alkyl-OPEs [81]. Cl-OPEs demonstrate broad-spectrum toxicity including neurotoxicity, endocrine disruption, immunotoxicity, and carcinogenicity, with TCPP and TDCIPP showing greater effects than TCEP despite structural similarities [82,83,84]. These concerns have led to regulatory action. The classification of TCEP and TDCIPP as Group II carcinogens by the European Commission [48]. Aryl-OPEs similarly show considerable cytotoxicity, neurotoxicity, reproductive toxicity, and immunotoxicity [81]. In contrast, alkyl-OPEs, while capable of inducing various toxic effects, generally exhibit weaker potency or require higher exposure concentrations to produce adverse outcomes comparable to those of Cl- or aryl-OPEs [81]. Furthermore, in terms of developmental toxicity, alkyl-OPEs primarily inhibit embryonic growth and cause abnormal heart rates, whereas aryl- and Cl-OPEs not only inhibit growth but also disrupt normal embryonic development, leading to malformations and more severe developmental defects [81].

The biotransformation of OPEs can significantly alter their toxicity profiles. Studies have shown that the degradation of TPHP in a bioelectrochemical system has been associated with decreasing acute toxicity to *Vibrio fischeri*, with luminescence inhibition decreasing from 17.6% to 4.4% after 72 h of incubation, indicating that the degradation process contributed to detoxification even though DPHP was present [85]. Similarly, for TCPs (denoted as ∑_3_-tricresyl phosphate (TCP), comprising TpCP, TmCP, and ToCP), their corresponding diesters (DmCP, DpCP, and DoCP) exhibited lower ROS production and cell apoptosis rates in A549 cells, indicating that the transformation of triesters to diesters for TCPs is a biological detoxification process [86]. This pattern is further supported by studies on alkyl-OPEs. Aerobic degradation of TBOEP and TNBP resulted in marked decreases in acute toxicity to *Vibrio fischeri*, with luminescence inhibition below 2.9% after degradation [17]. The lower toxicity of degradation products compared to precursor compounds can be attributed to their lower Log Kow values and higher water solubility [17]. However, recent studies have identified that hydroxylated transformation products, such as mono-hydroxylated phosphate (OH-TPHP), bis(2-butoxyethyl) hydroxyethyl phosphate (BBOEHEP), and bis(2-butoxyethyl) 3-hydroxyl-2-butoxyethyl phosphate (3-OH-TBOEP), act as strong endocrine disruptors via nuclear receptors, indicating that while overall toxicity may decrease during degradation, specific metabolic pathways may generate products with distinct toxicological concerns [17]. Furthermore, toxicity assessment of rimethyl phosphate (TMP) degradation in an electrogenic respiration system revealed that although most biodegradation intermediates showed lower acute and chronic toxicity to fish and green algae compared to TMP, certain intermediates (B2 and D2) exhibited slightly increased toxicity to Daphnia [87]. Notably, in some cases such as the degradation of isodecyl diphenyl phosphate (IDDP) and bis-(2-ethylhexyl)-phenyl phosphate (BEHPP), products including I4, I5, I6, B6, and B7 still possess higher acute and chronic toxicity than their parent compounds [88].

**Figure 3:**
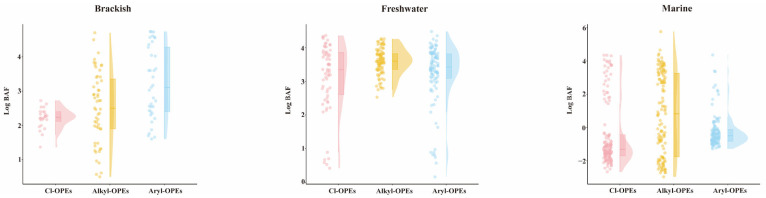
Bioaccumulation factors (BAFs) of differently classified OPEs in environmental media. pink for chlorinated OPEs (Cl-OPEs), yellow for alkyl-OPEs, and blue for aryl-OPEs.

In addition to these toxicity profiles, the ecological risks of OPEs are further amplified by their bioaccumulation potential in food webs. Bioaccumulation and biomagnification of OPEs occur across diverse ecosystems, with bioaccumulation factor (BAF) values varying significantly depending on the characteristics and chemistry of the different food webs [89,90]. Estuarine, freshwater, and marine matrices have shown varying magnification of Cl-OPEs, alkyl-OPEs, and aryl-OPEs (Figure 3). Notably, compared to brackish and freshwater environments, BAFs exhibit a wider range in marine. Differences in the relative concentrations of OPEs in species across environments are linked to site-specific sources and compound properties [90,91].

In summary, the toxicity of OPEs is intrinsically structure-dependent, with Cl- and aryl-OPEs exhibiting greater potency than alkyl-OPEs. Although biotransformation generally reduces acute toxicity through the formation of more polar diesters, the generation of specific hydroxylated intermediates and the persistence of certain high-toxicity transformation products underscore the need for comprehensive risk assessments targeting both parent compounds and their environmentally relevant metabolites.

## 4. Microbial Remediation Strategy

### 4.1. Degradation Schemes

OPEs exhibit high stability under typical wastewater treatment processes [48]. Once released into the environment, these compounds can undergo biotransformation through microbial degradation under both aerobic and anaerobic conditions [56,92]. The microorganisms that handle OPEs degradation are listed in Table S2. OPEs degraders are predominantly Proteobacteria at the phylum level. Specifically, *Sphingobium* exhibits potential degradation in OPEs (including TCEP, TDCIPP (Cl-OPEs), TBP (alkyl-OPEs), TPHP, and TCP (aryl-OPEs)) [93,94,95]. *Serratia odorifera* shows a high temperature adaptability in degrading TBP (4–37 °C) [96]. Species of *Dehalococcoides*, *Brevibacillus brevis*, *Nocardioidaceae* and *methanogens* are frequently found in OPE-contaminated environments [56,97,98]. The existence of these OPE degraders allows in situ bioremediation of OPE-polluted environments.

The biotransformation of OPEs occurs through multiple mechanisms, including hydrolysis, oxidation, hydroxylation, alkylation, and dealkylation, especially hydrolysis serving as a particularly crucial biotransformation pathway [84,99]. These compounds typically undergo simultaneous hydrolysis and hydroxylation, with the resulting intermediates subject to further transformation by methyltransferases [17].

#### 4.1.1. Cl-OPEs

Most Cl-OPEs can undergo microbial degradation through processes such as reduction, hydrolysis, and oxidation [15,56]. However, aerobic and anaerobic conditions lead to different degradation pathways for Cl-OPEs to be mineralized to inorganic phosphate [100]. In anaerobic environments, the biological dechlorinated of Cl-OPEs primarily occurs through electron transfer processes in the respiratory chain, involving microbiologically mediated redox reaction [100]. Specifically, the main conversion pathway for chlorinated organic compounds under anoxic conditions is reductive dechlorination [100]. The anaerobic microbial transformation pathways of TCEP include one-electron transfer and radical rearrangement [101]. The C-O bond cleavage results in the formation of bis(2-chloroethyl) phosphate (BCEP) and ethylene [101]. Then, the ethylene is reduced to ethane [101]. Notably, TCEP can undergo microbial degradation under anaerobic conditions through different pathways. Biotransformation products bis(2-chloroethyl) 2-hydroxyethyl phosphate (TCEP-OH) and 2-chloroethyl bis(2-hydroxyethyl) phosphate (TCEP-2OH) indicate that TCEP may be hydrolytically dechlorinated to form these intermediates, with BCEP further hydrolyzing to produce 2-chloroethyl (2-hydroxyethyl) hydrogen phosphate (BCEP-OH) [56]. Additionally, bis (2-chloroethyl) (2-oxoethyl) ester (TCEP-CHO) and phosphoric acid bis(2-chloroethyl) (arboxymethyl) ester (TCEP-COOH) were identified as microbial transformation products of TCEP through oxidation [15]. TCEP-OH is oxidized to TCEP-CHO and further oxidized to TCEP-COOH in the receiving waters of WWTPs under aerobic conditions. The molecular structure of TCPP consists of a phosphorus backbone and three chlorinated isopropyl groups, presenting the phosphoric center, C-Cl bonds, and -CH_3_ groups as the main cleavage sites for intermediate formation [84]. The microbial degradation of TCPP begins with a phosphotriesterase-catalyzed attack on the central phosphate, cleaving one oxygen-chlorinated isopropyl arm to produce C_6_H_13_C_l2_PO_4_ and an intermediate-C_3_H_5_Cl [84]. Similarly, the other oxygen-chlorinated isopropyl arm of C_6_H_13_C_l2_PO_4_ degrades to produce C_3_H_8_ClPO_4_, which is then oxidized to C_3_H_8_ClPO_4_ [84]. Therefore, the key biotransformation pathways for TCPP are hydrolysis and hydroxylation [84]. Additionally, TDCIPP can be hydrolyzed to yield phosphate and 1,3-dichloro-2-propanol (1,3-DCP), which is subsequently converted to glycerol through dehalogenation of 1,3-DCP [95].

#### 4.1.2. Alkyl-OPEs

The biotransformation of alkyl-OPEs involves several crucial steps: o-dealkylation, hydroxylation, oxidation, and conjugation, all of which enhance the hydrophilicity of the products and facilitate their removal [46]. Hydroxylation preferentially occurs at the third carbon atom due to its increased stability compared to other positions [17]. Under aerobic conditions, TBOEP is frequently detected in bacterial enrichment cultures [92]. The biotransformation of TBOEP begins with hydroxylation at either an internal or terminal carbon of the butoxyethyl group, resulting in the formation of four hydroxy tris(2-butoxyethyl) phosphate (TBOEP-OH) isomers [92]. TNBP degrades under aerobic conditions to produce various transformation products, including DNBP and dibutyl-3-hydroxybutyl phosphate (3-OH-TNBP) [17]. Additionally, dibutyl methoxybutyl phosphate (C_13_H_31_O_6_P) forms via the methoxylation of TNBP [17]. The degradation of TEHP occurs through O-ethylhexanol, leading to 2-ethylhexanol, and eventually producing dioctyl phosphate and monooctyl phosphate [102]. Notably, dialkyl phosphate esters (DAPs) emerge as the predominant transformation products during aerobic biotransformation of alkyl-OPEs [17].

#### 4.1.3. Aryl-OPEs

TPHP undergoes hydrolysis via ester bond cleavage, resulting in the formation of diphenyl phosphate (DPHP) and monophenyl phosphate (MPHP) [103]. Subsequent steps include hydroxylation and methylation, during which hydroxyphenyl-diphenyl phosphate (OH-DPHP) is produced through the hydroxylation of TPHP, and CH_3_-TPHP is generated via methylation [103]. During the biotransformation of TPHP, glutathionylation and glycosylation leading to the formation of novel derivatives such as glutathione-conjugated GSH-TPHP and monoglucosylated G-O-TPHP [103]. Additionally, dihydroxybenzene is produced through the further hydroxylation of phenol [103]. These findings indicate that the microbial degradation of TPHP involves processes, including hydrolysis, hydroxylation, methylation, glycosylation, and glutathione conjugation [103]. EHDPP undergoes hydrolysis through two pathways: one pathway generates 2-ethylhexyl monophenyl phosphate (EHMPP), followed by the release of phenol, while the other pathway produces monophenyl phosphate (MPHP) through the release of 2-ethylhexanol [104]. These phosphomonoesters are further hydrolyzed to produce phosphoric acid, phenol, and 2-ethylhexanol [104]. Hydrolysis and hydroxylation are the primary biodegradation processes mediated by rhizosphere microorganisms during the biotransformation of EHDPP, TPHP, and BEHPP, which share similar metabolic pathways [105]. TCP undergoes hydrolysis to form dicresyl phosphate (DCrP), which is subsequently hydrolyzed to produce cresol and dimethylphenol [104]. Alternatively, DCrP can undergo methylation to produce CH_3_-DCrP, which also leads to cresol and dimethylphenol through hydrolysis [104]. Additionally, mono-cresyl phosphate (MCrP) is another intermediate that can be hydrolyzed to form phosphoric acid and cresol or methylated to produce CH_3_-MCrP [104]. These findings underscore the complexity of TCP biotransformation, which involves multiple hydrolysis and methylation steps. The biotransformation of TCP primarily entails reductive dechlorination and hydrolytic dechlorination under anaerobic conditions [106]. Reductive dechlorination produces 5,6-dichloro-2-pyridinol, which undergoes further hydrolytic dechlorination to form 2,5,6-trihydroxypyridine [106]. Subsequent cleavage of the pyridine ring generates intermediates such as maleamide semialdehyde, maleamic acid, fumaric acid, and pyruvic acid. These processes illustrate the intricate pathways involved in TCP degradation [106].

The primary mechanism for the biotransformation of alkyl-OPEs and aryl-OPEs involves the cleavage of specific bonds by aerobic microorganisms [17]. The common microbial degradation pathways of alkyl-OPEs and aryl-OPEs include hydrolysis, hydroxylation, metathesis, and substitution [17]. For both Cl-OPEs and aryl-OPEs, particularly TCPP, TPHP, EHDPP, and BEHPP, hydrolysis and hydroxylation are crucial processes in their biotransformation [84]. The biodegradability of the three OPEs categories varies significantly under aerobic and anaerobic conditions. From the compiled data, four key patterns emerge: (i) substituent groups govern degradation efficiency, with aryl-OPEs consistently showing the highest aerobic degradability (>82%) followed by alkyl-OPEs (75–89.9%) and Cl-OPEs (32.5–100% but requiring long adaptation), (ii) oxygen availability determines the dominant hydrolysis/oxidation mechanism under aerobic conditions versus reductive dechlorination under anaerobic conditions, (iii) strain-specific degradation capabilities often exceed phylogenetic relatedness, as exemplified by different *Xanthobacter* strains showing markedly different TCEP degradation abilities, and (iv) synthetic consortia consistently outperform single strains, achieving higher degradation rates and greater mineralization (>75%) than pure cultures. These patterns provide a framework for selecting class-specific bioremediation strategies.

Based on these patterns, the most promising microorganisms and mechanisms for practical application can be identified for each OPE class: For Cl-OPEs, *Dehalococcoides*-containing consortia represent the most promising candidates for anaerobic bioremediation, achieving transformation of TCEP and TCPP within 10 days via reductive dechlorination [56,101]. Electron donor supplementation (e.g., acetate, lactate) is essential to sustain this activity [107]. For alkyl-OPEs, *Klebsiell* and *Rhodococcus* strains are highly effective under aerobic conditions, with degradation rates following first-order kinetics and half-lives ranging from 8.7 to 208 h [17,102]. Glucose amendment can further enhance degradation efficiency [17]. For aryl-OPEs, synthetic consortia combining *Sphingobium* and *Rhodococcus* achieve the highest performance, with half-lives as short as 4.53 h for TPHP and over 75% mineralization within one week [104,107,108]. These consortia maintain high efficiency across a broad range of pH (6–10), temperature (20–40 °C), and salinity (0–6%), making them suitable for diverse environmental conditions [108].

Overall, synthetic consortia represent the most promising strategy for practical OPE bioremediation, as they combine complementary degradation capabilities (e.g., dephosphorylation coupled with dehalogenation) and achieve higher mineralization rates than single strains. However, the choice of bioremediation approach must be tailored to site-specific conditions: anaerobic reductive dechlorination is recommended for Cl-OPEs in anoxic sediments, while aerobic bioaugmentation is more suitable for alkyl- and aryl-OPEs in environments.

Environmental conditions significantly influence OPE degradation patterns. Cl-OPEs demonstrate greater stability in marine environments, compounds such as TCEP and TCPP degrade more rapidly than high-molecular-weight alkyl-OPEs in sediments [80]. This variation indicates that the three OPEs groups demonstrate distinct degradation rates across different environmental matrices (water, sediment, and marine atmosphere) [80].

Notably, the degradation kinetics of OPEs vary considerably with their structural classes, as evidenced by the half-life data compiled from diverse environmental matrices (Table S1). Under aerobic conditions, chlorinated OPEs exhibit the longest persistence, with TCEP showing a half-life of approximately 60 h (2.5 days) in water, while TCPP and TDCIPP demonstrate half-lives of 1440 h (60 days) and 180–360 h (7.5–15 days), respectively. This recalcitrance correlates with the electron-withdrawing effect of chlorine substituents, which stabilizes the phosphate ester bond against nucleophilic attack and requires extended microbial adaptation periods for effective degradation [107].

In contrast, alkyl-OPEs and aryl-OPEs exhibit substantially shorter half-lives under aerobic conditions. For instance, TBOEP and TEHP show water half-lives of 208 h and 8.7 h, respectively, while TNBP and triisobutyl phosphate (TiBP) display half-lives of approximately 208 h and 15 h [17,109]. Aryl-OPEs such as TPHP and EHDPP exhibit water half-lives of 37.5 h and 15 h, respectively. The higher biodegradability of non-chlorinated OPEs is attributed to the greater susceptibility of their ester bonds to enzymatic hydrolysis, with degradation rates generally following the order: aryl-OPEs > alkyl-OPEs > Cl-OPEs under aerobic conditions [17].

Anaerobic conditions significantly alter this hierarchy. While triphenyl ester OPEs maintain relatively high removal rates (degradation time 50% (DT_50_) 4.3–6.9 days), chlorinated OPEs require substantial adaptation periods, with reported DT_50_ values of 18.4 days for TCEP and 10.0 days for TDCIPP upon electron donor supplementation [17]. Alkyl OPEs, however, exhibit minimal anaerobic degradability regardless of treatment conditions [17]. This oxygen-dependent degradation pattern reflects the distinct rate-limiting steps governing each OPEs class, with reductive dechlorination dominating Cl-OPEs transformation under oxygen-limited conditions [56], while hydrolysis and oxidation prevail for alkyl- and aryl-OPEs under aerobic conditions [17,107].

To provide a critical quantitative comparison of biodegradation performance across the three OPEs classes, the key differences in degradation efficiency, primary pathways, typical half-lives, and key degrading genera under both aerobic and anaerobic conditions are summarized in Table 1, with detailed strain level data compiled in Table S2.

Data compiled from Table S2 and references [17,56,84,95,103,104,107]. Degradation efficiencies vary with specific experimental conditions (strain type, initial concentration, incubation time, etc.), values shown represent reported ranges.

**Figure 4:**
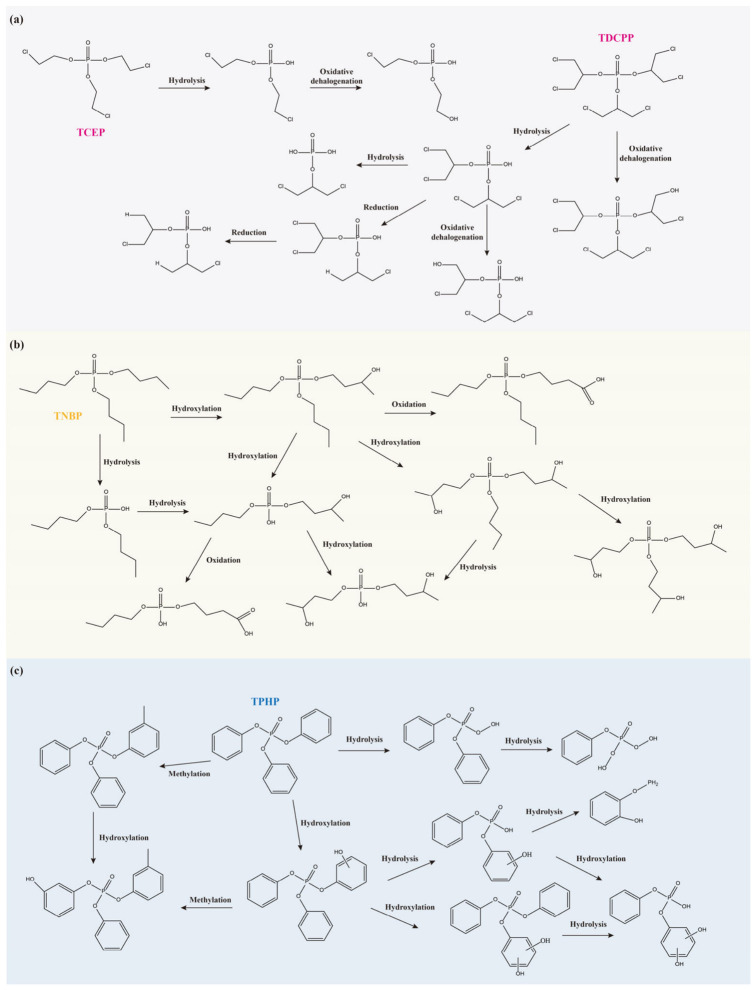
Schematic comparison of microbial degradation pathways for Cl-, alkyl-, and aryl-OPEs. (**a**) Cl-OPEs (TCEP and TDCIPP); (**b**) Alkyl-OPEs (TNBP); (**c**) Aryl-OPEs (TPHP).

To provide a visual summary of the class-specific degradation mechanisms discussed above, a schematic comparison of the main microbial degradation pathways for Cl-, alkyl-, and aryl-OPEs is presented in Figure 4.

As summarized in Table 1, the overall degradation trend under aerobic conditions follows the order: aryl-OPEs > alkyl-OPEs > Cl-OPEs, whereas anaerobic conditions shift the hierarchy to aryl-OPEs > Cl-OPEs > alkyl-OPEs. This oxygen-dependent reversal underscores the distinct rate-limiting steps governing each OPE class: reductive dechlorination facilitates Cl-OPEs transformation under oxygen-limited conditions, while hydrolysis and oxidation dominate the degradation of alkyl- and aryl-OPEs under aerobic conditions [17,56,107].

#### 4.1.4. Environmental Risks of Biotransformation Products

The environmental risks of OPEs cannot be fully assessed without considering the toxicity and fate of their biotransformation products, which may exhibit persistence and toxicity comparable to or even exceeding those of their parent compounds [17,81].

Diester metabolites, such as diphenyl phosphate (DPHP), di-n-butyl phosphate (DNBP), and bis(2-chloroethyl) phosphate (BCEP), are generally more polar and mobile than their parent triesters, potentially posing greater risks to aquatic ecosystems due to their prolonged environmental persistence [17,85]. Moreover, certain hydroxylated intermediates, including mono-hydroxylated TPHP (OH-TPHP), bis(2-butoxyethyl) hydroxyethyl phosphate (BBOEHEP), and bis(2-butoxyethyl) 3-hydroxyl-2-butoxyethyl phosphate (3-OH-TBOEP), have been identified as potent endocrine disruptors via nuclear receptor-mediated pathways, indicating that specific metabolic pathways may generate products with distinct toxicological concerns [17].

Importantly, the toxicity of degradation products does not always decrease relative to the parent compounds. While the aerobic degradation of TBOEP, TNBP, and TPHP resulted in markedly decreased acute toxicity to *Vibrio fischeri* (luminescence inhibition below 2.9% after degradation), and TPHP degradation in bioelectrochemical systems reduced luminescence inhibition from 17.6% to 4.4%, certain transformation products exhibit higher toxicity [17,85]. For example, intermediates generated during the degradation of isodecyl diphenyl phosphate (IDDP) and BEHPP still possess higher acute and chronic toxicity than their parent compounds [88]. Additionally, although most TCP isomers showed reduced toxicity after biotransformation, some degradation intermediates exhibited slightly increased toxicity to Daphnia [87].

These findings underscore that the ecological risks of OPEs should be assessed based on both parent compounds and their environmentally relevant transformation products, rather than focusing solely on the removal of the original contaminants [17,88]. Future studies should prioritize the identification, quantification, and toxicity characterization of OPE degradation intermediates to enable comprehensive risk assessments.

The compiled evidence reveals three distinct scenarios that clarify when biodegradation reduces environmental risk and when it leads to the formation of more hazardous intermediates.

Scenario 1: Clear detoxification. The aerobic degradation of TBOEP, TNBP, and TPHP consistently reduces acute toxicity to *Vibrio fischeri*, with luminescence inhibition falling below 2.9% after degradation [17]. Similarly, TPHP degradation in bioelectrochemical systems reduces luminescence inhibition from 17.6% to 4.4% [85]. In these cases, biotransformation to more polar diesters lowers bioavailability and toxicity.

Scenario 2: Formation of persistent or endocrine-disrupting intermediates. Hydroxylated transformation products of TPHP (OH-TPHP), TBOEP (3-OH-TBOEP), and other OPEs act as potent endocrine disruptors via nuclear receptor-mediated pathways [17]. In addition, diester metabolites such as DPHP, DNBP, and BCEP exhibit greater mobility and prolonged environmental persistence than their parent compounds [17,85]. Thus, while acute toxicity may decrease, chronic endocrine-disrupting effects can persist or even intensify.

Scenario 3: Increased toxicity of degradation products. In the degradation of IDDP and BEHPP, certain intermediates (I4, I5, I6, B6, and B7) exhibit higher acute and chronic toxicity than their parent compounds [88]. Likewise, some TCP degradation intermediates show increased toxicity to *Daphnia* [87].

These scenarios underscore that biodegradation should not be equated with detoxification. Risk assessments must consider both parent compounds and their transformation products, and remediation strategies should prioritize pathways that achieve complete mineralization rather than partial transformation.

### 4.2. Factors Affecting OPEs Biodegradation

**Figure 5:**
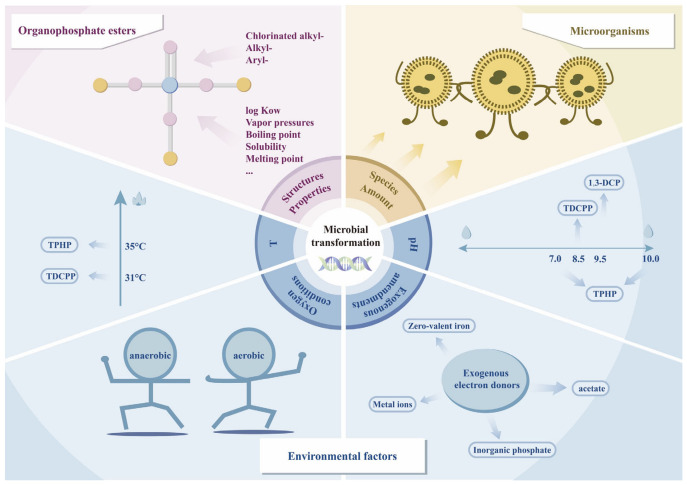
Critical factors influencing the microbial degradation of OPEs (T represents temperature). Pink for oxygen atoms, yellow for functional groups, and blue for phosphorus atoms.

The microbial transformation of OPEs is governed by three interacting key factors: the structural characteristics of OPEs themselves, the composition and adaptation of microbial communities, and prevailing environmental conditions (Figure 5). Understanding how each factor influences biodegradation is essential for predicting OPEs’ fate and designing remediation strategies.

#### 4.2.1. OPEs Structural Characteristics

The intrinsic molecular structure of OPEs fundamentally determines their bioavailability and susceptibility to enzymatic attack [99]. Structural features such as alkyl chain length, chlorine substitution patterns, and aryl ring configurations influence not only the initial degradation rates but also the predominant transformation pathways. For instance, the presence of chlorine atoms in Cl-OPEs contributes to their resistance to hydrolysis and oxidative cleavage, while the ester bonds in alkyl- and aryl-OPEs are more readily targeted by microbial enzymes. These structure-dependent differences underscore the need for class-specific investigations into OPEs’ biodegradation mechanisms.

#### 4.2.2. Microbial Community Composition

The presence and activity of specialized degrading microorganisms are prerequisite for efficient OPE biodegradation. Studies on microbial transformation typically employ microbial communities sourced from OPE-contaminated environments, including activated sludge, landfill leachate, e-waste sites, and polluted sediments [84,86,104,107]. These environments naturally enrich for microorganisms with adaptive capabilities. Specific bacterial taxa have been identified as key degraders for different OPE classes: *Burkholderiales* and *Rhizobiales* dominate in TCEP-contaminated microcosms, while *Sphingomonans*, *Nocardioides*, and *Streptomyces* contribute to TNBP degradation in anoxic soils [15,16]. The composition and diversity of these microbial communities directly determine the metabolic potential and degradation efficiency of the ecosystem.

#### 4.2.3. Environmental Conditions

Environmental parameters modulate both microbial activity and the physicochemical behavior of OPEs, thereby governing biodegradation kinetics and pathways.

Oxygen availability represents a master variable controlling OPE biodegradation. Aerobic and anaerobic conditions select for distinct microbial metabolisms and drive different degradation mechanisms. Under aerobic conditions, oxidative enzymes initiate electrophilic attacks, whereas anaerobic environments favor reductive and hydrolytic transformations. Previous studies indicate that alkyl-OPEs exhibit higher degradability under aerobic conditions compared to anoxic or anaerobic environments, a trend also observed for Cl-OPEs [107,110]. Notably, TCP biodegradation deviates from this pattern, demonstrating faster degradation under aerobic conditions than in anaerobic environments [106]. This exception is attributed to the ability of aerobic bacteria to undergo fermentation in oxygen-depleted soil environments, despite growth limitations under anaerobic conditions.

Temperature and pH significantly influence enzymatic activities and microbial growth. Strain TCM1 achieves maximum dephosphorylation of TDCIPP (2.53 μmol h^−1^ OD_660_^−1^) at 30 °C, while strain PY1 exhibits peak dehalogenation of 1,3-DCP (1.31 μmol h^−1^ OD_660_^−1^) at 35 °C [111]. Optimal pH conditions vary among strains: TCM1 shows maximum TDCIPP dephosphorylation at pH 8.5, whereas strain PY1′s dehalogenation activity peaks at pH 9.5 [111]. The TPHP-degrading strain YC-MTN exhibits strong pH dependency, with degradation rates exceeding 88.62% under neutral to alkaline conditions (pH 7.0–10.0) compared to only 6.20% in acidic environments (pH 6.5) [104].

Initial OPE concentration exerts a dual effect on biodegradation. Sufficient concentration is necessary to induce degrading enzymes and support microbial growth, but excessive concentrations may cause toxicity. Studies with TCEP demonstrated enhanced biodegradation at 10 μg/g compared to 5 μg/g, suggesting that higher concentrations promote enrichment of TCEP-degrading microorganisms in landfill soils [100]. For TCPP, concentrations below 1 mg/L limit bacteria-pollutant contact, while concentrations exceeding 1 mg/L inhibit bacterial growth and metabolic activity, resulting in decreased degradation rates [84].

Exogenous amendments can stimulate or suppress OPEs biodegradation through multiple mechanisms. Acetate supplementation altered microbial community composition in landfill soils but unexpectedly suppressed TCEP transformation [100]. Inorganic phosphates show compound-specific effects: while NaH_2_PO_4_ minimally impacted Cl-OPEs (TCEP and TDCIPP) degradation, it significantly enhanced microbial dehalogenation capacity, with optimal activity at 0.2 mM [112]. Similarly, KH_2_PO_4_ promoted bacterial growth and enhanced TCPP biotransformation [84]. Glucose effects vary by system: it facilitated high degradation rates (89.9%) for TPHP, TBOEP, and TNBP in mixed cultures, but showed no enhancement for aryl-OPEs degradation by strain YC-MTN, suggesting these compounds serve as preferred carbon sources for this strain [72,104].

However, direct comparisons of degradation efficiencies and kinetic parameters across studies remain challenging due to considerable variability in experimental design and environmental contexts. Four primary sources of inter-study differences merit critical consideration: (i) initial OPEs concentrations, with higher concentrations often inhibiting microbial activity or inducing alternative pathways, (ii) inoculum sources, as communities from contaminated sites exhibit faster degradation due to prior functional gene enrichment, (iii) substrate specificity and co-metabolism, where the presence of supplementary carbon sources (e.g., glucose) can significantly enhance degradation efficiency, and (iv) analytical methods, where differences in detection limits and reporting formats complicate direct comparisons. These factors highlight the need for standardized protocols and caution when interpreting data from different sources.

In summary, OPE biodegradation results from complex interactions between compound structure, microbial community composition, and multiple environmental parameters. Understanding these interconnected factors is essential for predicting OPEs’ fate in natural systems and developing effective bioremediation strategies.

### 4.3. Enzymatic Degradation of OPEs

Enzymatic activities mediate the biodegradation of organic pollutants, reflecting the complex interactions between microbial community structure and environmental conditions [84,86]. Structurally similar microcontaminants may be catalyzed by the same enzymatic systems, highlighting the importance of understanding enzyme diversity and function in OPE biodegradation [113].

To illustrate the enzymatic degradation of OPEs, the phosphotriesterase (PTE) family serves as a representative example. PTEs are binuclear metallohydrolases capable of hydrolyzing phosphorus ester bonds in a broad range of organophosphate compounds [114]. The most extensively characterized PTE was initially identified from *Flavobacterium* sp. ATCC 27551 and *Pseudomonas diminuta* MG, with the *opd* gene encoding this enzyme [115,116]. A closely related variant, *opdA* from *Agrobacterium radiobacter* P230, shares 90% amino acid sequence identity with the *Flavobacterium* enzyme but exhibits enhanced activity toward dimethyl organophosphates [117]. Successful trials have demonstrated that PTE-expressing strains can achieve rapid detoxification of organophosphates in contaminated water and soil matrices, with degradation efficiencies exceeding 90% within hours [117].

Beyond PTEs, a diverse array of enzymatic systems contributes to OPE degradation across the three compound classes. For Cl-OPEs, reductive dehalogenases (encoded by *cbrA* and *rdhA*) mediate the cleavage of C-Cl bonds under anaerobic conditions, as demonstrated in *Dehalococcoides*-enriched cultures that transformed TCEP and TCPP within 10 days [56,101]. For alkyl-OPEs, cytochrome P450 monooxygenases initiate hydroxylation and oxidative dealkylation of compounds such as TBOEP and TNBP, representing the primary phase I metabolic step [17,102]. For aryl-OPEs, glutathione-S-transferases facilitate phase II detoxification through glutathione conjugation, as documented for TPHP, EHDPP, and BEHPP [103]. Additionally, alkaline phosphatases (*phoA*, *phoD*) contribute to the hydrolysis of phosphomonoester intermediates, with *phoD* identified as a key phosphatase gene in OPEs-contaminated agricultural soils [15,97]. This enzyme repertoire collectively covers the three OPEs categories through distinct yet complementary mechanisms: reductive dechlorination for halogenated compounds, oxidative cleavage for alkyl chains, and hydrolytic or conjugative pathways for aryl esters.

Microbial transformation of organophosphorus compounds primarily occurs through the hydrolysis of phosphate groups, catalyzed by enzymes such as organophosphate hydrolases and phosphotriesterases [99]. The phosphoesterase family of enzymes plays specific roles in OPEs degradation: phosphotriesterase catalyzes TiBP hydrolysis, phosphodiesterase facilitates diisobutyl phosphate (DiBP) breakdown, and phosphomonoesterase catalyzes the cleavage of phosphomonoester bonds [112]. Alkaline phosphatase plays a crucial role in hydrolyzing structurally simple alkyl phosphates and facilitates mono-chloroethyl phosphate (MCEP) hydrolysis in TCEP microcosms [15,113]. Additional hydrolytic enzymes, including acid phosphatase and haloalkylphosphorus hydrolase, contribute to the degradation process through various hydrolytic mechanisms [15,101].

For alkyl-OPEs, the primary degradation pathways involve organophosphorus hydrolase, paraoxonase, and cytochrome P450 [102]. Under anoxic conditions, reductive dechlorination serves as a key biodegradation pathway for chlorinated organic compounds such as TCEP. This process involves *cbrA*-encoded chlorobenzene-reductive dehalogenase [101]. Additionally, haloacid dehalogenase catalyzes the hydrolytic dechlorination of C-Cl bonds in halogenated pollutants [56].

Glutathione-S-transferases (GSTs) play a significant role in the transformation of aryl-OPEs, particularly TPHP, EHDPP, and BEHPP [103]. As phase II xenobiotic metabolizing enzymes, GSTs mediate the colocalization of hydrophobic xenobiotic components with glutathione [118]. The glutathione thiol group forms conjugates with xenobiotic electrophilic molecules, facilitating their subsequent metabolism, excretion, and detoxification [118]. Additional enzymatic pathways involve various monooxygenases (including cytochrome P450, flavin-dependent, soluble di-iron, and alkane monooxygenases), methyltransferase, and urease, further diversifying the microbial transformation mechanisms of OPEs [92,103].

The nature of microbial transformation primarily relies on the roles of extracellular and intracellular enzymes. Studies have shown that both enzyme types efficiently degrade TCPs, with intracellular enzymes showing superior performance [86]. For instance, intracellular enzymes from strain ZY1 exhibited faster degradation rates than intact cells, indicating that cell membrane transport may be a rate-limiting step in whole-cell degradation [86]. Similarly, the intracellular enzymes of O. tritici readily cross the cell membrane and can effectively degrade TEHP [102]. In contrast, Providencia rettgeri showed comparable TCPP degradation efficiency between intracellular and extracellular enzymes, achieving 28.4–31.2% decomposition within 12 h [84].

Under aerobic conditions, oxygenase activity increases significantly, promoting oxidative reactions [107]. In contrast, anaerobic environments exhibit 40–75% higher reductase and protease activities compared to aerobic conditions, while dehydrogenase activities show a 40–60% increase [107]. These elevated enzyme activities under anaerobic conditions facilitate hydrolysis and reduction processes, significantly influencing OPE degradation pathways and efficiency.

OPE pollutants and their metabolites can induce specific enzyme activities, facilitating the degradation and mineralization of both parent compounds and their transformation products [113]. During TiBP biodegradation, the activities of several key enzymes increase significantly, including hydroxylase, dehydrogenase, phosphomonoesterase, phosphodiesterase, and phosphotriesterase [113]. Hydroxylase serves as the primary enzyme responsible for the hydroxylation of TiBP and its metabolite DiBP, with its induction directly influencing the degradation efficiency of alkyl-OPEs [98]. Dehydrogenase, an oxidoreductase enzyme, plays a crucial role in soil biological activity [98]. Its increased activity results from microbial stimulation by OPEs and root secretions that serve as supplementary carbon sources [98]. Studies have shown enhanced dehydrogenase activity following the addition of sodium acetate, TiBP, and its metabolites (DiBP and mono-isobutyl phthalate (MiBP)) [113]. The significant increase in phosphomonoesterase activity indicates its crucial role in enhancing TiBP and metabolite degradation [113].

High OPE concentrations can inhibit enzyme activity through multiple mechanisms, including masking catalytically active sites, denaturing protein structures, and competing with natural enzyme substrates [98]. Additionally, catalase, a key oxidoreductase in biodegradation, exhibits a biphasic response to TCPP and TPHP exposure [98]. This tetrameric enzyme demonstrates reduced activity under high OPEs concentrations due to potential ferroportin translocation, compromising the enzyme’s active centers [98]. The complex interaction between OPE concentration and enzyme activity highlights the importance of maintaining optimal conditions for effective biodegradation.

Beyond the functional characterization of enzymes, recent advances in molecular biology have begun to elucidate the genetic underpinnings of OPE biodegradation. The most extensively studied organophosphate-degrading genes are *opd* (organophosphate degradation) and its homologue *opdA*, which encode phosphotriesterases (PTEs) capable of hydrolyzing a broad range of organophosphate triesters [119]. The *opd* gene was initially identified in *Flavobacterium* sp. ATCC 27551 and *Brevundimonas diminuta* MG, while *opdA* from *Agrobacterium radiobacter* P230 shares 90% amino acid sequence identity with organophosphate hydrolase but exhibits enhanced activity toward dimethyl organophosphates [117,119]. Both enzymes belong to the amidohydrolase superfamily and contain a binuclear metal center (typically Zn^2+^) at their active sites, which is essential for catalytic activity [107].

For chlorinated OPEs, reductive dehalogenase genes (*cbrA* homologues) have been identified in *Dehalococcoides*-enriched cultures, with expression levels significantly upregulated upon TCEP exposure [56]. These enzymes mediate the cleavage of C-Cl bonds under anaerobic conditions, representing a distinct degradation mechanism from the hydrolytic pathways of phosphotriesterases.

Metagenomic and metatranscriptomic approaches have recently enabled the identification of key degraders and their functional genes in complex microbial communities. Liang et al. (2023) applied metagenomic sequencing to a TBOEP-degrading enrichment culture and reconstructed 14 metagenome-assembled genomes (MAGs), revealing that *Rhodococcus ruber* strain C1 was the most active degrader, with upregulation of various monooxygenase, dehydrogenase, and phosphoesterase genes throughout the degradation process [92]. Similarly, Cheng et al. (2025) identified *phoA*, *phoB*, *phoD*, and *glpQ* as the key phosphatase-encoding genes responsible for OPEs’ ester bond cleavage in agricultural soils, with *Nocardioides* and *Pimelobacter* as the core degrading genera [97].

These genetic insights provide a foundation for enzyme engineering and bioaugmentation strategies. For instance, site-directed mutagenesis of organophosphate hydrolase at residues H254 and H257 has been shown to alter substrate specificity, with mutants exhibiting up to 18-fold higher activity toward soman analogues [120]. The expression of *opd* genes in heterologous hosts, including *E. coli* and *Pichia pastoris*, has also been successfully achieved, enabling high-yield production of recombinant OPE-degrading enzymes for potential industrial applications [121].

Future research should prioritize the elucidation of regulatory mechanisms governing OPE-degrading gene expression, particularly under nutrient-limited conditions where OPEs may serve as alternative phosphorus sources. Metagenomic and metatranscriptomic analyses, combined with functional validation, will be essential for identifying novel degradation genes and enzymes from uncultured microorganisms, thereby expanding the toolbox for bioremediation of OPE-contaminated environments.

## 5. Conclusions and Future Perspective

This review systematically examines the classification, properties, environmental occurrence, toxicity, and microbial remediation strategies of three major OPE classes (Cl-OPEs, alkyl-OPEs, and aryl-OPEs). Our analysis reveals distinct environmental behaviors among OPE classes: Cl-OPEs demonstrate greater persistence (sediment half-lives: 654–1621 days) and long-range transport potential, while alkyl-OPEs and aryl-OPEs exhibit higher hydrophobicity, leading to preferential sediment partitioning and stronger bioaccumulation. Toxicological impacts are structure-dependent: Cl-OPEs and aryl-OPEs generally show greater toxicity than alkyl-OPEs, with effects spanning neurotoxicity, endocrine disruption, and carcinogenicity. Microbial degradation offers promising remediation potential, with key degrading genera including *Sphingobium*, *Serratia*, and *Dehalococcoides*, and primary pathways involving hydrolysis, oxidation, hydroxylation, and dealkylation. Enzymatic mechanisms include phosphotriesterases, dehalogenases, cytochrome P450, and glutathione-S-transferases.

Despite these advances, several critical knowledge gaps remain that hinder the practical application of microbial remediation for OPE-contaminated environments:

### 5.1. Key Scientific Gaps

(1) Pathway elucidation in complex matrices: While degradation pathways for individual OPEs have been characterized under laboratory conditions using pure cultures, the transformation fate of OPEs and their intermediates in complex environmental matrices (e.g., mixed-contaminated soils, sediments with varying organic carbon content, and estuarine waters with fluctuating salinity) remains poorly understood. The interactive effects of co-occurring pollutants on OPEs degradation pathways have been rarely investigated.
(2) Quantitative structure–biodegradability relationships (QSBR): Current understanding of how specific substituent patterns (e.g., chlorine number and position, alkyl chain length, aryl ring substitution) influence degradation rates is largely qualitative. Robust QSBR models that can predict the environmental persistence of NOPEs based on their molecular descriptors are urgently needed to prioritize chemicals for risk assessment without exhaustive experimental testing.
(3) Enzyme catalytic mechanisms and substrate specificity: Although several OPE-degrading enzymes have been identified, the structural basis of their substrate specificity and catalytic efficiency remains largely unresolved. High-resolution crystal structures of key enzymes (e.g., phosphotriesterases complexed with OPE substrates) are lacking, limiting rational enzyme engineering efforts to enhance degradation efficiency and broaden substrate range.
(4) Toxicity of transformation products: The environmental risks of OPEs cannot be fully assessed without considering their degradation intermediates. Many transformation products (e.g., hydroxylated TPHP, diesters) exhibit endocrine-disrupting activity or greater mobility than parent compounds, yet systematic toxicity data for these metabolites remain scarce. Integrated risk assessment frameworks that account for both parent compounds and their transformation products are urgently needed.

Despite these gaps, the compiled data reveal several consistent patterns that inform future research: (i) degradation efficiency follows a structure-dependent hierarchy (aryl-OPEs > alkyl-OPEs > Cl-OPEs under aerobic conditions), (ii) oxygen availability determines the dominant degradation mechanism, (iii) synthetic consortia consistently outperform single strains, and (iv) biodegradation does not universally equate to detoxification. These findings provide a foundation for prioritizing the research directions outlined below.

### 5.2. Practical Approaches for Field Application

The selection of appropriate remediation strategies must be guided by the environmental occurrence characteristics of OPEs in target matrices. As compiled in Table S1, different OPEs classes exhibit distinct environmental distribution patterns that directly inform degradation strategy design. Cl-OPEs (e.g., TCEP, TCPP, TDCIPP), with typical water half-lives of 60–1440 h and sediment half-lives of 542–38,900 h, are frequently detected in surface water, sediment, and WWTP effluents (Table S1). Their persistence in anoxic sediments necessitates anaerobic bioremediation approaches, particularly electron donor supplementation to stimulate reductive dechlorination by *Dehalococcoides*-containing consortia [56,107]. In contrast, alkyl-OPEs (e.g., TBOEP, TNBP, TEHP) exhibit shorter water half-lives (8.7–208 h) and are predominantly found in indoor air, WWTP effluents, and biota (Table S1), favoring aerobic bioaugmentation strategies using *Klebsiella* or *Rhodococcus* strains [17,107]. Aryl-OPEs, characterized by high logKow values (4.51–5.73) and strong sediment partitioning, are prevalent in house dust, sediment, and biota (Table S1); their high aerobic biodegradability (>82%) makes them suitable candidates for bioaugmentation with *Sphingobium* or *Rhodococcus* in aerobic bioreactors or constructed wetlands [104,107]. For marine environments, where salinity limits many conventional degraders, halotolerant genera such as *Roseobacter* (Table S2), which achieve 100% degradation of TPHP and TCP, offer promising bioaugmentation candidates [104]. Thus, the environmental occurrence data summarized in Table S1 are not merely descriptive but serve as a critical foundation for designing matrix-specific, efficient degradation strategies tailored to the contaminant profile and physicochemical conditions of each environmental compartment.

To translate laboratory findings into effective remediation strategies, the following practical approaches should be prioritized:

(1) Bioaugmentation with specialized degrader consortia: Rather than single strains, microbial consortia with complementary degradation capabilities (e.g., *Sphingobium* for dephosphorylation combined with *Arthrobacter* for dehalogenation) should be developed and tested under field-relevant conditions. Immobilization of these consortia on carrier materials (e.g., biochar, zeolite) could enhance their survival and activity in contaminated soils and sediments.
(2) Biostimulation of indigenous degraders: The addition of electron donors (e.g., acetate, lactate) has been shown to stimulate reductive dechlorination of Cl-OPEs under anaerobic conditions. Similarly, oxygen release compounds or periodic aeration could promote aerobic degradation of alkyl- and aryl-OPEs. Optimizing nutrient amendments (nitrogen, phosphorus, and carbon sources) based on site-specific conditions represents a cost-effective bioremediation approach.
(3) Enzyme-based remediation technologies: Immobilized enzyme systems (e.g., phosphotriesterases encapsulated in alginate beads or immobilized on magnetic nanoparticles) offer advantages over whole-cell approaches, including higher stability, reusability, and reduced mass transfer limitations. However, the cost-effective production of recombinant OPE-degrading enzymes and their long-term activity under field conditions require further development.
(4) Constructed wetlands and phytoremediation: Integrated systems combining plants (e.g., ryegrass, rice) with rhizosphere microorganisms have shown potential for OPE removal. Future studies should optimize plant–microbe combinations, hydraulic retention times, and substrate materials to enhance OPE removal efficiency while minimizing the accumulation of toxic transformation products.
(5) Monitoring and assessment tools: Sensitive and field-deployable analytical methods (e.g., portable mass spectrometry, biosensors based on OPEs-degrading enzymes) are needed for real-time monitoring of OPEs and their metabolites during remediation. Additionally, molecular tools (e.g., qPCR targeting *opd*, *cbrA*, and *phoD* genes) could serve as proxies for degradation potential in contaminated sites, guiding the selection of appropriate remediation strategies.

### 5.3. Outlook

Future perspectives on OPE pollution should extend beyond end-of-pipe bioremediation toward holistic and sustainable mitigation strategies. A critical preventive approach lies in the design of inherently safer alternatives. For instance, the development of bio-based or halogen-free flame retardants with lower persistence and toxicity can reduce the long-term environmental burden, aligning with the principles of green chemistry. Furthermore, the industrial phase-out of hazardous OPEs should be accompanied by systematic green solvent screening—as demonstrated by the replacement of chloroform and dichloromethane with acetone, ethanol, and water in electrospinning processes—to avoid technological lock-in and unintended health risks during manufacturing [122]. Such preventive measures, combined with advances in material science and predictive environmental risk assessment, offer a pathway toward sustainable production consumption systems.

Addressing these scientific gaps and advancing practical remediation approaches requires interdisciplinary collaboration among microbiologists, environmental chemists, engineers, and risk assessors.

Beyond single-strain isolation, recent studies have increasingly focused on microbial consortia for OPE degradation, which offer distinct advantages over pure cultures through interspecies cooperation and metabolic partitioning. For instance, a synthetic consortium composed of *Rhodococcus* sp. YC-JH2 and *Sphingopyxis* sp. YC-JH3 at a 1:1 ratio was constructed for aryl-OPE degradation. Under optimum conditions (pH 8, 35 °C), the consortium utilized aryl-OPEs as the sole carbon source and achieved rapid degradation with half-lives of 4.53 h for TPhP, 21.11 h for TCP, and 23.0 h for EHDPP, with over 75% mineralization within one week [108]. The consortium maintained high efficiency across a broad range of pH (6–10), temperature (20–40 °C), and salinity (0–6%), and tolerated TPhP concentrations up to 500 mg/L without inhibition. Similarly, a SynCom composed of *Bacillus subtilis*, *Bacillus licheniformis*, and *Ralstonia pickettii* was constructed during sludge composting under ∑_7_OPEs stress [123]. This consortium achieved nearly complete removal (>93%) of TCPP and TPhP, with hydrolysis identified as the preferentially initiated degradation pathway. Functional enzymes such as phosphatases, along with bacterial strains from *Rhodococcus* and *Paracoccus*, synergistically participated in the degradation process, and SynCom addition promoted ∑7OPEs removal by boosting phosphomonoesterase activity and enriching phosphatase-producing microorganisms, with pH and total phosphorus identified as critical environmental factors influencing this degradation [123]. These findings highlight that synthetic or enriched consortia, rather than single strains, represent a more promising strategy for practical OPEs bioremediation, particularly in complex matrices such as wastewater treatment systems, composting facilities, and marine environments. Future research should prioritize: (i) rational design of consortia based on complementary degradation pathways (e.g., dephosphorylation coupled with dehalogenation), (ii) elucidation of interspecies interactions and quorum sensing mechanisms that enhance overall degradation activity, (iii) validation of SynCom performance under field-relevant conditions (variable temperature, pH, salinity, and co-contaminants), (iv) integration of metagenomic and metatranscriptomic tools to monitor functional gene expression and community stability during bioremediation, and (v) validating bioremediation technologies at pilot and field scales. Such consortia-based approaches, combined with cell immobilization technologies, offer a viable path toward scalable and robust OPE remediation in real-world environments.

## Figures and Tables

**Table 1:** Comparative summary of biodegradation performance across three OPE classes.

OPEs Class	Representative Compounds	Key Degrading Genera	Aerobic Degradation Efficiency	Anaerobic Degradation Efficiency	Primary Degradation Pathways	Typical Water Half-Lives
Cl-OPEs	TCEP, TCPP, TDCIPP	*Sphingobium*, *Dehalococcoides*, *Xanthobacter*, *Burkholderia*	Low to moderate (32.5–100%; 2 h to 5 days, strain-dependent; requires long adaptation)	Moderate (DT_50_ 10.0–18.4 days with electron donors)	Reductive dechlorination, hydrolysis, oxidation	60–1440 h
Alkyl-OPEs	TBOEP, TNBP, TEHP, TBP	*Klebsiella*, *Rhodococcus*, *Ochrobactrum*, *Serratia*, *Sphingomonas*	High (75–89.9%; first-order k = 0.314/h for TBOEP)	Minimal (negligible degradation regardless of treatment)	Hydrolysis, hydroxylation, O-dealkylation, oxidation	8.7–208 h
Aryl-OPEs	TPHP, EHDPP, TCP	*Sphingobium*, *Sphingopyxis*, *Rhodococcus*, *Brevibacillus*, *Roseobacter*	High to very high (up to 100% for optimized consortia; 29–100% for single strains, 24 h to 7 d)	Moderate to high (DT_50_ 4.3–6.9 days)	Hydrolysis, hydroxylation, methylation, glutathionylation	15–37.5 h
Overall Trend			Aryl-OPEs > Alkyl-OPEs > Cl-OPEs	Aryl-OPEs > Cl-OPEs > Alkyl-OPEs		

## Data Availability

No new data were created or analyzed in this study. Data sharing is not applicable to this article.

## Supplementary Materials

The following supporting information can be downloaded at https://www.mdpi.com/article/10.3390/toxics14070618/s1, Table S1: Classification and properties of OPEs; Table S2: Classification and characteristics of microorganisms that degrade OPEs [124,125,126,127,128,129,130,131,132,133,134,135,136,137,138,139,140,141,142,143,144,145,146,147,148,149,150,151,152,153,154,155,156,157,158,159,160,161,162,163,164,165,166,167,168,169,170,171,172,173,174,175,176,177,178,179,180,181,182,183,184,185,186,187,188,189].

## Abbreviations

BDP	bisphenol A bis (diphenyl phosphate)
BEHPP	bis-(2-ethylhexyl)-phenyl phosphate
BFRs	brominated flame retardants
CDP	cresyl diphenyl phosphate
Cl-OPEs	chlorinated organophosphate esters
DAPs	dialkyl phosphate esters
DiBP	diisobutyl phosphate
DIN	dissolved inorganic nitrogen
DNBP	dibutyl phosphate
DPHP	diphenyl phosphate
DT_50_	degradation time 50%
EHDPP	2-ethylhexyl diphenyl phosphate
ELV	end-of-life vehicle
GSTs	glutathione-S-transferases
IDDP	isodecyl diphenyl phosphate
LRAT	long-range atmospheric transport
MAGs	metagenome-assembled genomes
MCEP	mono-chloroethyl phosphate
MiBP	mono-isobutyl phosphate
MPHP	monophenyl phosphate
NOPEs	novel organophosphate esters
OPEs	organophosphate esters
PBDEs	polybrominated diphenyl ethers
POM	particulate organic matter
PTE	phosphotriesterase
PU	polyurethane
QSBR	quantitative structure–biodegradability relationships
RDP	resorcinol diphosphate
TBP	tri-n-butyl phosphate
TBOEP	tris(2-butoxyethyl) phosphate
TCEP	tris(2-chloroethyl) phosphate
TCIPP	tris(1-chloro-2-propyl) phosphate
TCP/TCrP	tricresyl phosphate
TCPP	tris(2-chloro-1-methylethyl) phosphate
TDCIPP	tris(1,3-dichloro-2-propyl) phosphate
TEHP	tri(2-ethylhexyl) phosphate
TEP	triethyl phosphate
TiBP	triisobutyl phosphate
TN	total nitrogen
TNBP	tri(n-butyl) phosphate
TPHP	triphenyl phosphate
WWTPs	wastewater treatment plants
